# Qualification of Membrane Filtration for Planetary Protection Flight Implementation

**DOI:** 10.3389/fmicb.2022.871110

**Published:** 2022-04-29

**Authors:** Kristina Vaikovna Stott, Lyssa Morgan, Caitlin Shearer, Morgan Byrd Steadham, Mihaela Ballarotto, Ryan Hendrickson

**Affiliations:** ^1^Jet Propulsion Laboratory, California Institute of Technology, Pasadena, CA, United States; ^2^Johns Hopkins University Applied Physics Laboratory, Laurel, MD, United States

**Keywords:** planetary protection, membrane filtration (MF), NASA Standard Assay, spores, *Bacillus atrophaeus*

## Abstract

Planetary protection is the practice of preventing forward and backward contamination of solar system bodies. Spacecraft and associated surfaces are sampled to ensure compliance with bioburden requirements. Current planetary protection sampling and processing methodologies consist of extracting microbial cells from wipe or swab samples through a procedure (NASA Standard Assay) that includes sonication, heat shock, and pour-plate steps. The pour-plate steps are laborious and prolonged. Moreover, results can be imprecise because only a fraction of the sample fluid is plated for CFU enumeration (80% for swabs and 25% for wipes). Thus, analysis requires that a pour fraction extrapolation factor be applied to CFU counts to account for bioburden in the remaining sample volume that is not plated. This extrapolation results in large variances for data, decreasing the accuracy of spore bioburden estimation of spacecraft hardware. In this study, we investigated the use of membrane filtration as an alternative method to pour-plate processing. Membrane filtration is an appealing methodology for planetary protection because it can process greater sample volumes and reduces the data variance for bioburden enumeration. A pour fraction extrapolation factor is still applied for both swabs and wipes (92%), however, it is a greater pour fraction than the pour-plate method. Here we present data collected by the Jet Propulsion Laboratory and the Applied Physics Laboratory to experimentally determine the equivalency of membrane filtration to pour-plate methodology for implementation during the NASA Standard Assay. Additionally, we outline the planned procedures for two membrane filtration systems: Pall^®^ Laboratory Manifold system and Milliflex^®^ Plus Vacuum Pump System. Both systems demonstrated equivalence of the membrane filtration method to the pour-plate method.

## Introduction

Planetary protection is a field concerned with the responsible exploration of space (Meltzer, 2012). Extraterrestrial life detection has been a key goal in the field of astrobiology. Per a white paper report from the Biosignatures Standards of Evidence Community Workshop, it is critical for the astrobiology community to develop a universal scientific framework for life detection claims to ensure clear and consistent communication with the scientific community and the public. If organisms were found on a celestial body, it would be necessary to determine the origin of that life – is it native to that extraterrestrial environment, or is it spacecraft-borne from Earth? In 1967, the United Nations Outer Space Treaty put forth guidelines to preserve the scientific integrity of future explorations (United Nations, 1967), namely in Article IX of the treaty. Since then, the Committee on Space Research has updated the guidelines that national space agencies can adopt as part of their mission planning (COSPAR, 2008). In compliance with the Committee on Space Research policies, the National Aeronautics and Space Administration (NASA) monitors spacecraft bioburden to minimize the inadvertent contamination of solar system planets or moons (forward contamination) (Committee on Preventing the Forward Contamination of Mars, National Research Council, 2006). Although spacecraft and their components are assembled in cleanroom facilities (Moissl et al., 2007), spore-forming microbes are likely to persist (Venkateswaran et al., 2003; La Duc et al., 2004a,b; Crawford, 2005; Osman et al., 2006; Satomi et al., 2006). For this reason, the NASA standard spore assay was developed to estimate the aerobic spore-forming organisms on spacecraft hardware surfaces, using a cultivation-dependent method, as described by NASA procedures and guidelines (NPG: 5340.1D).

The compilation of procedures for assessing the microbial burden on spacecraft hardware, including the NASA Standard Assay procedure, is found in NASA-HDBK-6022, Handbook for the Microbial Examination of Space Hardware (2010). Briefly, the NASA Standard Assay is a culture method designed to enumerate microbial burden from spacecraft that has been sampled *via* a sterile cotton swab or polyester wipe suitable for small and large hardware surfaces, respectively. Microbes present on these sampling devices are extracted during a sonication step. Samples are then heated to 80 ± 2°C, to target heat-shock resistant spores. Next, 2.0- or 4.0-mL portions of the sample fluid are aseptically pipetted into Petri dishes (also referred to as plates), and sterile molten trypticase soy agar (TSA) is added to each plate. Once the mixture is solidified, plates are incubated (32°C, 3 days), and Colony Forming Units (CFU) are counted at 24, 48, and 72 h.

Planetary Protection Engineers at the Jet Propulsion Laboratory (JPL) have extensively used the NASA Standard Assay (NSA) for flight missions, including the InSight and Mars Science Laboratory missions (Benardini et al., 2014; Hendrickson et al., 2020) as well as the recent Mars 2020 mission. While the method is effective, it is inefficient in terms of labor, since 2–4 plates must be generated per swab and 13–25 plates per wipe. Moreover, results can be imprecise because only a fraction of the sample fluid is plated for enumeration (80% for swabs and 25% for wipes). This requires that a pour fraction extrapolation factor be applied to CFU results to account for the remaining sample that is not plated.

Thus, for the current Europa Clipper mission, we investigated a more sustainable method, membrane filtration, as an alternative to the pour-plate method for processing samples. Membrane filtration (MF) has been extensively studied (Grinnell, 1929; Bowman et al., 1967; Blosse et al., 1998; Wang et al., 2008; Onyango et al., 2010), used in the healthcare, food, and water industries (Bordner et al., 1978; Solomon et al., 1978; Sharpe et al., 1979; Smith et al., 1993; Lee et al., 2007; Crittenden et al., 2012), and approved for use on European Space Agency missions (ECSS-Q-ST-70-55 Working Group, 2008). For planetary protection applications, MF is an attractive alternative because the entire sample volume can be processed, increasing the accuracy of spore burden estimation of spacecraft hardware. Moreover, compared with the pour-plate method, MF has an overall cost savings due to a reduction in labor and or materials.

Although the Office of Planetary Protection has approved MF for use, NASA-HDBK-6022 does not include a specific procedure. Here we describe the equipment, materials, processes, and analyses used to develop a membrane filtration protocol to effectively enumerate bioburden from spacecraft surfaces, comparable to the pour-plate method. Specifically, results in this paper describe two independent studies: Jet Propulsion Laboratory Biotechnology and Planetary Protection Group (using Pall^®^ Laboratory Manifold and Filter Funnels, Whatman^®^ 0.2 μm cellulose acetate filter, and standard Petri dish with TSA) and Johns Hopkins University Applied Physics Laboratory – Materials Engineering and Planetary Protection Section (using Milliflex^®^ Plus Vacuum Pump, Milliflex-100, 0.22 μm white gridded funnel and filter unit, and Prefilled Milliflex^®^ Culture Media Cassettes).

## Materials and Methods

### NASA Standard Assay

**FIGURE 1:**
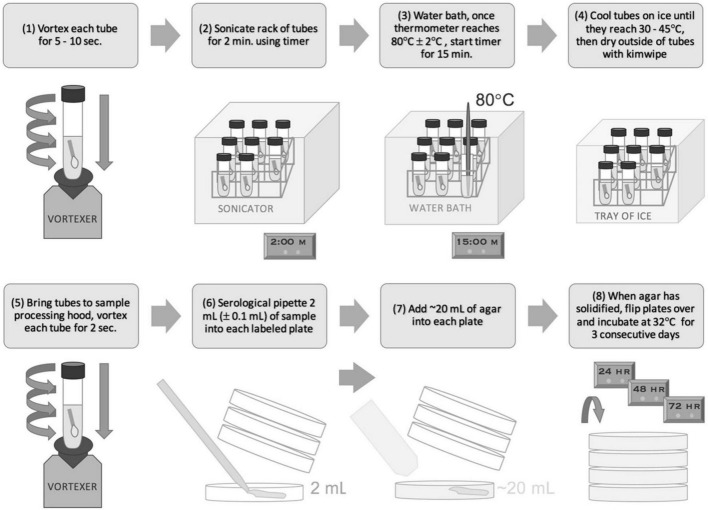
NASA Standard Assay for swab samples.

**FIGURE 2:**
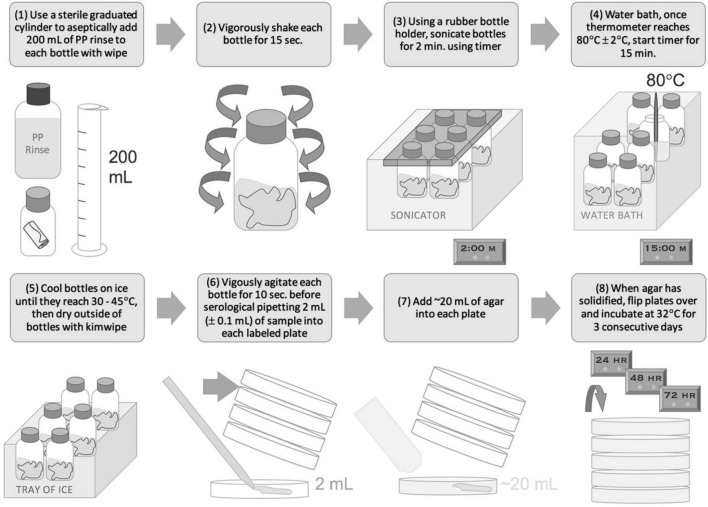
NASA Standard Assay for wipe samples.

Per NASA-HDBK-6022 (2010) samples are acquired using cotton swabs or 9′′ × 9′′ polyester wipes and processed *via* the associated NSA procedure. Both swab and wipe samples are premoistened with sterile DI water since they come in direct contact with flight hardware during sampling. As per NASA-HDBK-6022 (2010), swab samples are suspended in 10 mL 18.2 MΩ deionized water and wipes are suspended in 200 mL of planetary protection (PP) rinse solution for processing. PP rinse (buffered solution + Tween 80) was prepared in accordance with NASA-HDBK-6022 (2010). Samples then are processed *via* sonication for 2 min in an aqueous bath of 0.02% v/v Tween 80, at 19–27 kHz (Figures 1, 2). Samples then undergo “heat shock” at 80°C for 15 min to enable the detection of aerobic, mesophilic, and cultivable spores. Samples are then quickly cooled to 30–35°C. Portions (2 or 4 mL) of each sample are poured into sterile Petri dishes followed by a standard growth medium required by NASA-HDBK-6022 (2010), trypticase soy agar (TSA). The plates are incubated at 32°C for a period of 72 h, during which planetary protection engineers count colony forming units (CFUs) every 24 h.

### Preparation of Samples (Axenic Cultures; Jet Propulsion Laboratory)

*Bacillus atrophaeus* spores (∼1 μm) were obtained from Mesa Laboratories (#9372, Bozeman, MT, United States). A working spore stock solution was prepared containing 100 spores/mL. Following vortexing, 50 μL of the spore stock solution was transferred each into the three sample types: wipes, swabs, and control. Wipe samples refer to glass bottles containing only 200 mL of planetary protection (PP) rinse solution. PP rinse (buffered solution) was prepared in accordance with NASA-HDBK-6022 (2010). Swab samples refer to glass test tubes containing only 10 mL 18.2 MΩ deionized water. The wipe and swab sampling devices themselves (Polyester wipes, TX3211, Texwipe, Kernersville, NC, United States; Cotton Tipped Applicators, 806WC, Puritan, Guilford, ME, United States) were excluded during testing to minimize the potential variability of CFU recovery efficiency; the devices were previously validated by the Planetary Protection Office, thus we focused on comparing the methodologies only. The control samples refer to 50 μL of the working spore stock solution that was directly plated onto TSA plates.

### Preparation of Samples (Soil and *B. atrophaeus* Solutions; Applied Physics Laboratory)

*Bacillus atrophaeus* spores (∼1 μm) were obtained from Mesa Laboratories (#9372, Bozeman, MT, United States). *B. atrophaeus* has long been used as a model planetary protection organism as it grows well in the NSA incubation conditions (Aerobic, 32C, grows on TSA) and has a clear red pigmentation that can help distinguish it from potential contamination. Soil from APL’s campus was selected for its diverse, undefined, and abundant spore population instead of cleanroom samples that have a lower overall spore abundance and much higher variability in spore populations. Two types of solutions were made to directly compare NSA and MF: soil in DI water and *B. atrophaeus* in DI water. The soil solutions consisted of three trials (low, medium, and high concentrations, denoted as Soil concentration 1, 2, and 3, respectively) and the *B. atrophaeus* solutions consisted of one trial. Each trial consisted of one set of pour-plate samples, and two sets of membrane filtration samples for redundancy, denoted as “MF-APL 1” and “MF-APL 2,” each with 20 replicates. For the soil solutions, 1 g of dried soil was dried in open indoor air over the course of 1 week. It was then suspended in DI water and passed through a 20 μm filter to remove large solids. Spores were selected for by heat shocking the DI soil solution for 15 min at 80°C per NASA-HDBK-6022 (2010), thus assuming only spores remained in the soil samples after the heat shock. This starting solution was then serially diluted to a dilution factor of 1:10,000. For *B. atrophaeus* solutions, the *B. atrophaeus* spore stock was diluted to achieve approximately 1.25 spores/mL. Each sample consisted of 8 mL DI water to imitate a swab sample. A wipe-equivalent sample was not tested.

### Pour Plating

In accordance with the NASA Standard Assay, swab and wipe samples, as prepared in section “Preparation of Samples (Axenic Cultures; Jet Propulsion Laboratory)” or “Preparation of Samples (Soil and *B. atrophaeus* Solutions; Applied Physics Laboratory),” were sonicated, heat shocked, cooled, and processed *via* pour plating (NSA) (Figures 1, 2). For wipe samples, 4 mL was dispensed each into 12 Petri dishes and 2 mL was dispensed into 1 Petri dish, to total 50 mL of processed volume (PN 351029, Corning, Tewksbury, MA, United States) and filled with 1.5% TSA (Difco™ Tryptic Soy Agar PN 236920, BD Biosciences, Franklin Lakes, NJ, United States, or equivalent) using a peristaltic pump (Delta Scientific Medical Fill Master, or equivalent). For swab samples, 4 mL was dispended each into 2 Petri dishes and filled with TSA (alternatively 2 mL each into 4 Petri dishes). Plates were then incubated at 32°C and enumerated at 24, 48, and 72 h. Since only a fraction (50 mL for wipes and 8 mL for swabs) of the total sample volume (200 mL for wipes and 10 mL for swabs) is plated, the CFU enumerated at 72 h was multiplied by the pour fraction to extrapolate the final CFU. For wipe samples a pour fraction of 4× was applied to CFU results since 25% of the sample was processed (50/200 mL). For swab samples a pour fraction of 1.25× was applied to CFU results since 80% of the sample was processed (8/10 mL). A negative control consisting of a petri dish consisting of only poured TSA was included in each sample set to ensure no contamination occurred during the processing.

### Membrane Filtration (Pall Laboratory Manifold System; Jet Propulsion Laboratory)

The membrane filtration equipment includes a 3-place laboratory manifold with hose barb cap, end cap, and manifold valves (PN 4889, Pall, Port Washington, NY, United States). The equipment also includes manifold standard adapters (PN 4891, Pall, Port Washington, NY, United States), 150 mL magnetic filter funnels (PN 424, Pall, Port Washington, NY, United States), Whatman 0.2 μm cellulose acetate membrane filters (PN 10404112, Cytiva, Marlborough, MA, United States), forceps, 1/4′′ inner diameter Tygon E-3603 laboratory tubing (Saint-Gobain, Malvern, PA, United States), and a 1 L glass filter flask.

The membrane filtration setup was assembled in a laminar flow environment or Class II, Type A2 biological cabinet and consisted of the following: The Pall laboratory manifold was first connected to a filter flask using Tygon tubing and a rubber stopper. The barbed end of the filter flask was attached to a vacuum source *via* Tygon tubing. The 150 mL magnetic filter funnels were placed into each of the three manifold openings *via* the manifold standard adapters, and subsequently, the 0.2 μm Whatman filters were placed inside the magnetic filter funnels using sterile forceps.

**FIGURE 3:**
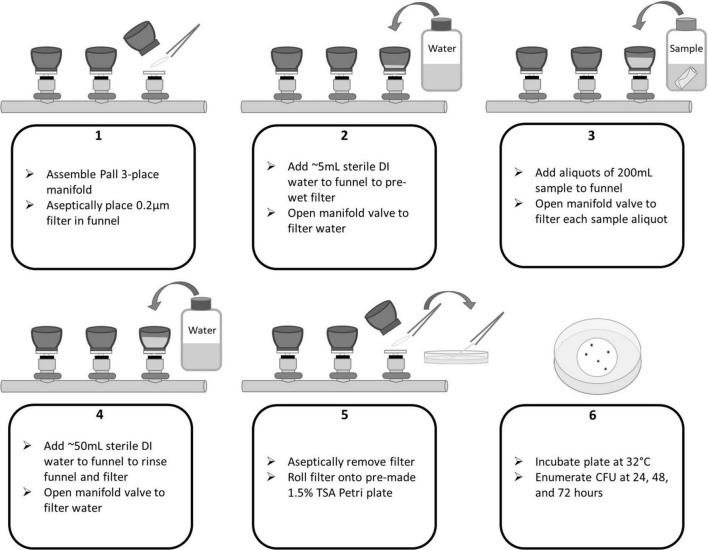
Pall Laboratory Manifold flow diagram for membrane filtration of wipe samples.

**FIGURE 4:**
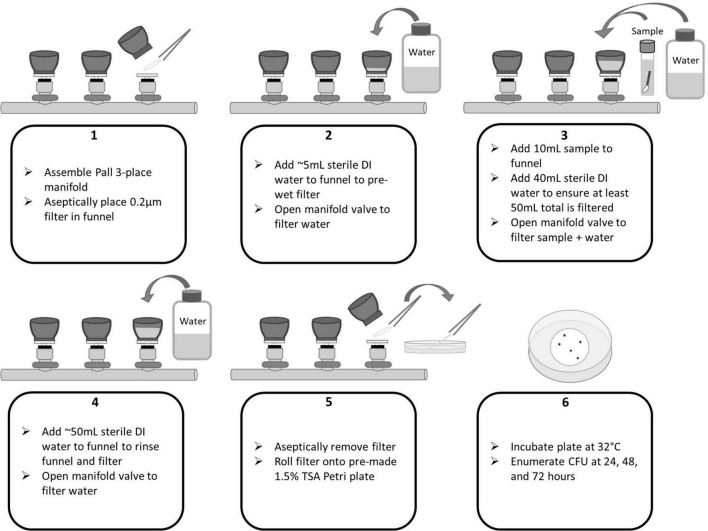
Pall Laboratory Manifold flow diagram for membrane filtration of swab samples.

In accordance with the NASA Standard Assay, swab and wipe samples were sonicated, heat shocked, and cooled. To begin membrane filtration (MF), 5 mL of sterile 18.2 MΩ deionized water was filtered through each funnel to wet the filters. Next, the entire volume of each sample (10 mL for swabs or 200 mL for wipes) was poured into a filter funnel. For swabs, an additional 40 mL DI water was added to ensure a minimum of 50 mL is filtered through the funnel. The volumes were filtered *via* vacuum and the sample allowed to drain completely. A negative control consisting of only sterile 18.2 MΩ deionized water was included in each sample set to ensure no contamination occurred during the processing. The funnels and filters were rinsed of potentially inhibitory residues with an additional ∼50 mL of sterile water followed by filtration. Using sterile forceps, the membrane filters were removed from the funnels and placed into sterile Petri dishes prepared with TSA by slowly rolling the filter onto the solidified agar to avoid trapped air bubbles underneath the filter. Plates were then incubated at 32°C and enumerated at 24, 48, and 72 h. See Figures 3, 4 for process flow diagram. Since the entire sample volume is filtered for both wipe and swab samples (in the absence of sampling devices), the CFU enumerated at 72 h represents the final CFU (i.e., the pour fraction is 100%).

### Membrane Filtration (Millipore Milliflex PLUS System; Applied Physics Laboratory)

The membrane filtration equipment includes a Milliflex Plus Pump Single Head Kit (PN MXPPLUS01, MilliporeSigma, Burlington, MA, United States), Milliflex-100, 100 mL, 0.22 μm white gridded funnel and filter in one (PN MXGSWG124, MilliporeSigma, Burlington, MA, United States), Milliflex Cassette prefilled with Tryptic Soy Agar (PN MXSMCTS48, MilliporeSigma, Burlington, MA, United States), and forceps.

The membrane filtration setup was assembled in a laminar flow environment and consisted of the following: the sterilized pump head is seated onto the pump and wiped down with 70% isopropyl alcohol (IPA). The system is turned on and “manual” filtration mode is selected. Using sterile forceps, the mesh spacer from a funnel is moved to the pump head. Next the funnel is pushed down over the mesh spacer and pump head. The clear cover is removed from the funnel and temporarily set aside.

**FIGURE 5:**
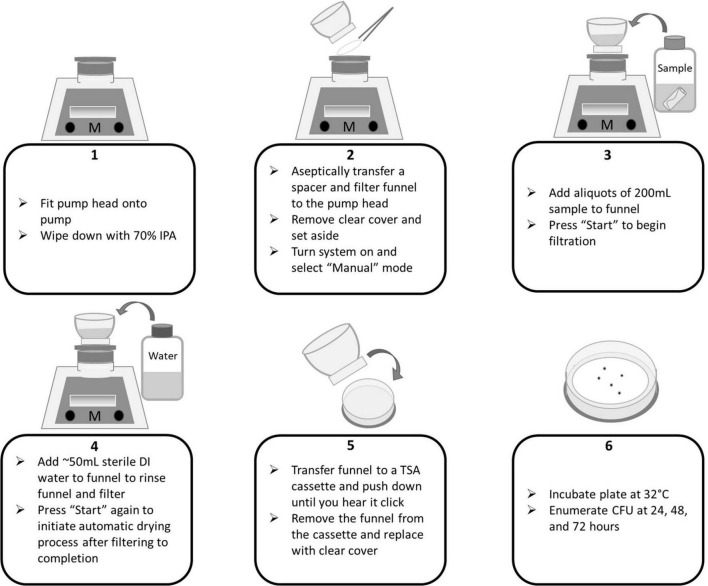
Milliflex flow diagram for membrane filtration of wipe samples.

**FIGURE 6:**
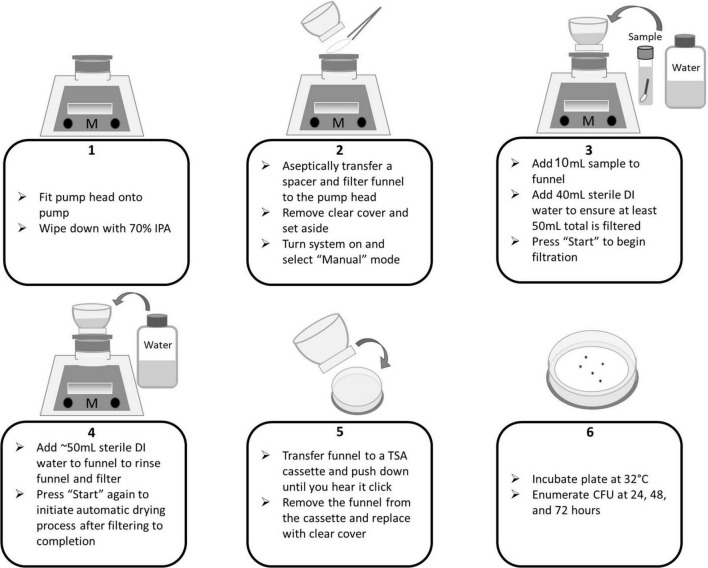
Milliflex flow diagram for membrane filtration of swab samples.

In accordance with the NASA Standard Assay, swab and wipe samples were sonicated, heat shocked, and cooled. To begin membrane filtration (MF), 8 mL of each sample was added to the funnel. For experiments conducted in this study, APL added 8 mL of sample instead of 10 mL. To ensure a minimum of 50 mL is filtered through the funnel, DI water was added. The “Start” button is pressed on the pump to initiate filtration. A negative control consisting of only sterile DI was included in each sample set to ensure no contamination occurred during the processing. The funnels and filters were rinsed of potentially inhibitory residues with an additional ∼50 mL of sterile water followed by filtration. The “Start” button was pressed again to initiate the automated drying process to remove excess moisture from the filter. Following filtration, the clear cover is returned to the funnel. The entire funnel assembly is removed from the pump head (mesh spacer is left behind) and fitted onto a TSA cassette by pushing down on the funnel assembly until a “click” is heard. The funnel is removed and the clear cover is moved to the cassette. Plates were then incubated at 32°C and enumerated at 24, 48, and 72 h. See Figures 5, 6 for process flow diagram. The entire sample volume (8 mL) was filtered (in the absence of sampling devices). Thus, the CFU enumerated at 72 h represents the final CFU (i.e., the pour fraction is 100%).

## Results

### Pall Laboratory Manifold System (Jet Propulsion Laboratory)

**FIGURE 7:**
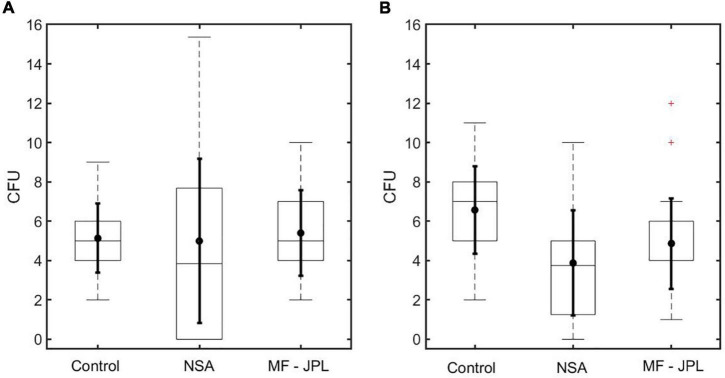
JPL CFU recovery results. Box plot marks the 25th and 75th percentiles. Solid dot in box plot marks the mean. Solid horizontal line in box plot marks the median. Dotted vertical line in box plot marks extreme points. Solid vertical line in box plot marks the standard deviation. Plus sign in box plot denotes outlier. **(A)** CFU recovery comparing NSA and MF-JPL for wipe samples. Results demonstrate similar median between control, NSA, and MF-JPL. Larger data range can be observed with NSA method due to pour fraction extrapolation. **(B)** CFU recovery comparing NSA and MF-JPL for swab samples. Results demonstrate similar median between NSA and MF-JPL. The control median was not similar to either the NSA or MF-JPL methods, likely due to the lack of surfactant since swab samples are submerged in water. Larger data range can be observed with NSA method due to pour fraction extrapolation. MF-JPL median is 4 (overlaps with bottom of box in box plot).

Colony forming units (CFU) recovery was compared between the two methods, NSA and MF, for wipe samples, using a known concentration of *B. atrophaeus* spores (Figure 7A and Table 1). Each method tested, and a control, consisted of thirty replicates. Non-parametric Mann–Whitney *U* analysis (significance level of 0.05) was conducted comparing the median CFU counts between methods. Mann–Whitney *U* test was utilized as data resulted in non-normal distribution indicating the need for a non-parametric test, as there is no assumption of normality or equal variances. The sample medians were statistically equivalent between each method, including the control (Table 2; *z* value for all method comparisons > critical value of −1.96 and <+1.96). The NSA method resulted in a considerably larger CFU recovery data range than the MF method or control, among which eight out of thirty replicates resulted in zero CFU after 72 h of incubation. These results are not surprising since, with the NSA method, only 50 out of 200 mL is plated; applying a pour fraction extrapolation factor (0.25) leads to greater variance, decreasing the accuracy of spore bioburden estimation. These results demonstrate that the NSA and MF methods are comparable in CFU recovery, but NSA has a higher variance. Moreover, MF allows for greater resolution of bioburden *via* processing of total volume compared with the NSA method.

**TABLE 1:** CFU recovery comparing NSA and MF (JPL).

Method	Mean (CFU)	Standard deviation (CFU)
**Wipe with PP rinse**		
Control	5.13	1.76
NSA	4.99	4.18
MF	5.40	2.16
**Swab with water**		
Control	6.57	2.21
NSA	3.88	2.67
MF	4.87	2.30

**TABLE 2:** Statistical equivalence comparing NSA and MF (JPL).

Sample condition	Mann–Whitney *U*: Value, Z crit = ±1.959 (alpha = 0.05)	Statistically equivalent?
**Wipe with PP rinse**		
Control vs. NSA	−0.798	Yes
Control vs. MF	−0.444	Yes
NSA vs. MF	−1.183	Yes
**Swab with water**		
Control vs. NSA	−3.813	No
Control vs. MF	−3.013	No
NSA vs. MF	−1.693	Yes

CFU recovery was compared between the two methods, NSA and MF, for swab samples, using a known concentration of *B. atrophaeus* spores (Figure 7B and Table 1). Each method tested, and a control, consisted of thirty replicates. A non-parametric Mann Whitney U analysis (significance level of 0.05) was also used to compare the median CFU counts between methods, because data resulted in non-normal distribution. The sample medians were statistically equivalent between the NSA and MF methods (Table 2; *z* value of −1.693 > critical value of −1.96 and <+1.96). However, the control median was not statistically equivalent to either the NSA (Table 2; *z* value of −3.813 < critical value of −1.96) or MF (Table 2; *z* value of −3.013 < critical value of −1.96) methods. This recovery loss in the NSA and MF methods could be due to the lack of surfactant since swab samples are submerged in water and not PP rinse like the wipes; the recovery efficiency may be reduced due to spores adhering to the side of the glass during processing, as no surfactant is present to decrease surface tension. The NSA method resulted in a larger CFU recovery data range than the MF method, among which three out of thirty replicates resulted in zero CFU after 72 h of incubation. These results are not surprising since, with the NSA method, only 8 out of 10 mL is plated; applying a pour fraction extrapolation factor (0.8) leads to greater variance (albeit less so than wipe samples), decreasing the accuracy of spore bioburden estimation. These results demonstrate that the NSA and MF methods are comparable in CFU recovery. Moreover, MF allows for greater resolution of bioburden *via* processing of total volume compared with the NSA method.

### Millipore Milliflex PLUS System (Applied Physics Laboratory)

**FIGURE 8:**
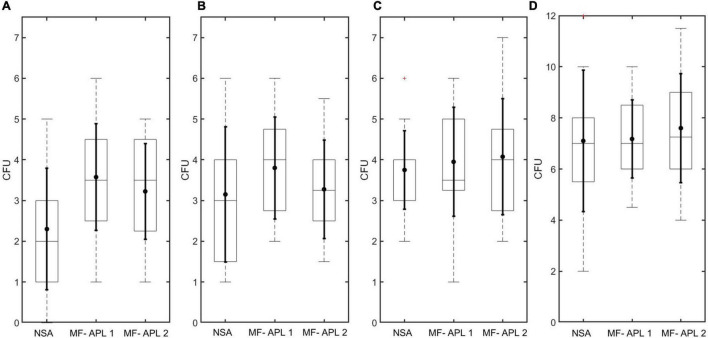
APL CFU recovery results. Box plot marks the 25th and 75th percentiles. Solid dot in box plot marks the mean. Solid horizontal line in box plot marks the median. Dotted vertical line in box plot marks extreme points. Solid vertical line in box plot marks the standard deviation. Plus sign in box plot denotes outlier. **(A–C)** CFU recovery comparing NSA and MF-APL for soil samples in 8 mL DI water for three different spore concentrations. **(D)** CFU recovery comparing NSA and MF-APL for *Bacillus atrophaeus* samples in 8 mL DI water.

CFU recovery was compared between the two methods, NSA and MF, for soil solutions using three different spore concentrations (low, medium, and high, denoted as soil concentration 1, 2, and 3, respectively). Results show that the MF method had higher mean CFUs than the NSA method (Figures 8A–C and Table 3). In two out of the three sample sets tested, the mean CFUs captured for the methods differed by less than one. *T*-Test was performed after evaluating the distribution of the differences between paired values, where the distribution of the differences should be approximately normally distributed for the *T*-Test to be implemented. For these sample sets, MF and NSA were found to be statistically equivalent (*p*-value > 0.05, Table 4). For one of the sample sets (concentration 1), the methods were found to not be statistically equivalent (*p*-value < 0.05) with MF collecting more CFUs. Altogether, the MF method is able to recover the same CFU number or more compared to the NSA method. MF and NSA perform similarly even at different concentrations. Moreover, the MF data generally had smaller standard deviation suggesting improved precision compared to NSA.

**TABLE 3:** CFU recovery comparing NSA and MF (APL).

Method	Spore source	Mean (CFU)	Standard deviation (CFU)
NSA	Soil concentration 1	2.300	1.4903
MF 1	Soil concentration 1	3.575	1.3106
MF 2	Soil concentration 1	3.225	1.1751
NSA	Soil concentration 2	3.150	1.6631
MF 1	Soil concentration 2	3.800	1.2503
MF 2	Soil concentration 2	3.275	1.2083
NSA	Soil concentration 3	3.750	0.9665
MF 1	Soil concentration 3	3.950	1.3367
MF 2	Soil concentration 3	4.075	1.426
NSA	*B. atrophaeus*	7.100	2.7701
MF 1	*B. atrophaeus*	7.175	1.5241
MF 2	*B. atrophaeus*	7.600	2.1312

**TABLE 4:** Statistical equivalence comparing NSA and MF (APL).

Sample condition	Spore source	*T*-test *P*-value	Statistically equivalent?
NSA vs. MF 1	Soil concentration 1	0.006	No
NSA vs. MF 2	Soil concentration 1	0.039	No
MF 1 vs. MF 2	Soil concentration 1	0.4407	Yes
NSA vs. MF 1	Soil concentration 2	0.141	Yes
NSA vs. MF 2	Soil concentration 2	0.809	Yes
MF 1 vs. MF 2	Soil concentration 2	0.212	Yes
NSA vs. MF 1	Soil concentration 3	0.6211	Yes
NSA vs. MF 2	Soil concentration 3	0.4021	Yes
MF 1 vs. MF 2	Soil concentration 3	0.7868	Yes
NSA vs. MF 1	*B. atrophaeus*	0.9028	Yes
NSA vs. MF 2	*B. atrophaeus*	0.4511	Yes
MF 1 vs. MF 2	*B. atrophaeus*	0.4214	Yes

CFU recovery was compared between the two methods, NSA and MF, for *B. atrophaeus* solutions. There was less than 10% difference in the mean CFUs counted for each method (Figure 8D and Table 3). MF and NSA methods were shown to be statistically equivalent (*p*-value > 0.05, Table 4).

### Membrane Filtration Pour Fraction Extrapolation Factor

In the method comparison studies performed by JPL and APL, we excluded the wipe and swab sampling devices themselves during testing to minimize the potential variability of CFU recovery efficiency. Thus, for MF experiments, the pour fraction was 100%. To determine the pour fraction of MF with the sampling devices included, we measured the final volume poured out from each vessel (glass test tubes containing swabs and glass media bottles containing wipes) compared with the initial volume (10 mL for swabs and 200 mL for wipes). From 36 replicates, the average final volume was 9.55 mL for swabs (standard deviation = 0.07) and 185.11 mL for wipes (standard deviation = 1.94). The remaining volume was contained in the sampling devices (∼0.45 mL for swabs and ∼14.89 mL for wipes) and was unable to be extracted during processing. Subtracting the standard deviation values from the average final volumes, we calculated a 95% pour fraction for swabs and a 92% pour fraction for wipes. Thus, for implementation of MF as part of the NASA Standard Assay, we recommend using a 92% pour fraction overall for simplicity for both swabs and wipes.

## Discussion

In both the JPL and APL data, we observed a lower mean in the NSA method compared to the MF method. This result may be explained by the fact that with the NSA method, a fraction of the sample volume is plated. When planetary protection engineers sample flight hardware, it is common to expect CFU in the lower ranges (0–5) given that flight hardware is routinely cleaned. With the NSA method, these lower CFU values can skew the mean. While an extrapolation factor is applied, this mathematically derived number may not represent an experimentally derived number. In other words, when only 25% of the sample volume for wipes, or 80% of the sample volume for swabs, is processed with the NSA, there is always the possibility that there may be ≥1 CFU in the un-plated suspension. The only way to obtain a true experimentally derived number is to process the entire sample volume; with MF, 0 CFU is more likely a “true” 0 than with the NSA method since the entire volume is plated with MF. Another possible explanation for the reduction in CFUs is the less-than-ideal oxygen availability in agar with pour-plate methods (Van der Meeren et al., 2001; Somerville and Proctor, 2013). Even though the agar layer is relatively thin, and some have reported the quick recovery of oxygen in agar medium at <1 cm post-autoclaving (Van der Meeren et al., 2001), the oxygen availability to microbes embedded in agar is far less than what is available to surface-grown microbes. As previously described, the growth conditions of the NSA result in an underestimation of overall microbes recovered (La Duc et al., 2007; Ghosh et al., 2010; Moissl-Eichinger et al., 2015; Hendrickson et al., 2021), however, this did not impact the results of this study as only equivalency was investigated.

Should planetary protection engineers elect to use MF as part of the NASA Standard Assay, they may need to vary the number of filters required for each sample based on the expected cleanliness of the sampled surfaces. For example, facility samples from surfaces that may be less rigorously cleaned than flight hardware (e.g., floors, benches, ground support equipment, etc.) may require two or more filters be used on a single wipe sample to prevent filter saturation or coalescing of colonies, for accurate CFU enumeration.

In our results, we reported the recovery loss in the swab samples when comparing NSA and MF with the control. This could be due to the lack of surfactant since swab samples are submerged in water and not PP rinse like the wipes. Future studies may focus on experimenting with swabs submerged in PP rinse and potentially changing the NASA Standard Assay to suspend swabs in PP Rinse instead of water, to optimize the recovery efficiency by reducing the possibility of spores adhering to the side of the glass walls of the sampling tubes during processing.

In our study, we elected to use a sterilizing grade filter (pore size of 0.2 μm). The industry standard for testing bacterial retention in any sterilizing grade filter is a recovery challenge of 1 × 10^7^ cfu/cm^2^ *Brevundimonas diminuta* bacteria (ASTM International, 2020) given Dr. Frances Bowman’s observation that this bacterium could repeatedly penetrate a 0.45 μm rated filter but not a 0.2 μm filter (Bowman et al., 1967; Duda et al., 2012; Ghuneim et al., 2018). However, the planetary protection engineer could elect to use a 0.45 μm filter (used in many applications for the microbiological examination of water, pharmaceuticals, and food) provided they perform testing similar to this study demonstrating equivalence with the NASA Standard Assay pour-plate method. In addition to considering a different pour size for the membrane filtration filter, future studies could also experiment with different filter materials (e.g., polyvinylidene fluoride or mixed cellulose esters).

The results from our study demonstrate that samples processed with MF result in equivalent bioburden detection capabilities with far less bioburden estimation variance than the NSA method. This was true for samples tested by JPL and APL, using both Pall^®^ Laboratory Manifold and Milliflex^®^ Plus Vacuum Pump Systems, respectively. MF is capable of processing a larger sample volume with less data variance, leading to a higher resolution of spore bioburden compared with the NSA method. Moreover, the MF method is a more sustainable option given the ability to process larger sample volumes; fewer plates means reduced TSA, cost, waste, and energy consumption.

## Data Availability Statement

The original contributions presented in the study are included in the article/supplementary material, further inquiries can be directed to the corresponding author/s.

## Author Contributions

KS contributed to JPL data interpretation, coordination with APL, and drafted the manuscript. LM contributed to JPL experimental design and laboratory experiments, statistical analysis, and initial chart development. RH contributed to JPL experimental design, coordination with APL, and data analysis and interpretation. CS and MS contributed to APL laboratory experiments. MB contributed to APL experimental design, coordination with JPL, and data analysis and interpretation. All authors contributed to reading and editing the manuscript, and approved the final manuscript.

## Conflict of Interest

The authors declare that the research was conducted in the absence of any commercial or financial relationships that could be construed as a potential conflict of interest.

## Publisher’s Note

All claims expressed in this article are solely those of the authors and do not necessarily represent those of their affiliated organizations, or those of the publisher, the editors and the reviewers. Any product that may be evaluated in this article, or claim that may be made by its manufacturer, is not guaranteed or endorsed by the publisher.

## Abbreviations

APL: Applied Physics Laboratory
CFU: colony forming units
JPL: Jet Propulsion Laboratory
MF: membrane filtration
NASA: National Aeronautics and Space Administration
NSA: NASA Standard Assay (as a method)
TSA: trypticase soy agar.
